# Does the Sharing of Resources Impact Health Among Married Couples? New Findings From Dyadic Models

**DOI:** 10.1093/geroni/igaa057.1941

**Published:** 2020-12-16

**Authors:** Shuangshuang Wang, Kyungmin Kim, Karen Lyons

## Abstract

As married couples aging together, their health behaviors and outcomes could be shaped by both one’s own and the spouse’s characteristics. Using dyadic datasets, speakers in this symposium explored the interdependence nature of marital relations by identifying the mechanisms of how shared resources or strains affect spouses’ physical and mental health outcomes among married couples. Wang, Kim, and Burr identified distinct types of personality configurations among older couples using the Health and Retirement Study, and examined how personality compatibilities could buffer negative effects of adverse life events on older couples’ mental health. Using data from the National Social Life, Health, and Aging Project, Proulx, Skoblow, and Han further investigated the associations between marital quality and mental health among caregiving dyads, with a special focus on a comparison of different caregiving groups (spouse, child, others). From a physical health perspective, Wilson and Novak presented the dynamic behind relationship quality, joint health behaviors, health problems, health satisfaction, and health similarity between spouses. Finally, Kim, Jang, Park, and Chiriboga focused on couple contexts for acculturation among older Korean immigrants in the U.S., and examined how each spousal acculturation level affects healthcare utilization and difficulties in health service use. Focusing on married couples, this symposium showcases the interplay of family experiences, health behaviors, and relational dynamics of both spouses in shaping their health, and highlights the benefits of dyadic approaches. Speakers and our discussant, Dr. Karen Lyons, will discuss implications for social program design and future research.

